# A Fatal Case of Isolated Renal Mucormycosis in an Immunocompetent Male

**DOI:** 10.7759/cureus.29593

**Published:** 2022-09-26

**Authors:** Kevin D Healey, Davong D Phrathep, Stefan Anthony, Michael A Jenkins, Lynda Gentchev, Ahmad O Rifai

**Affiliations:** 1 College of Osteopathic Medicine, Lake Erie College of Osteopathic Medicine, Bradenton, USA; 2 Urology, Advanced Urology Institute, Panama City, USA; 3 Pathology, HCA Florida Gulf Coast Hospital, Panama City, USA; 4 Nephrology, The Virtual Nephrologist, Lynn Haven, USA

**Keywords:** urology, nephrectomy, sepsis, immunocompetent, mucormycosis

## Abstract

Mucormycosis (MM) is an angio-invasive fungal infection that often presents in immunocompromised patients. Isolated renal MM is an uncommon presentation that has been documented as a life-threatening condition in immunocompetent patients due to its poor prognosis. Here, we present a rare case of isolated renal MM in a 27-year-old male who presented with left flank pain, nausea, and vomiting. Upon further investigation, a renal infarct was discovered, and he underwent a subsequent nephrectomy. A renal biopsy revealed MM. The patient’s infection spread, and he ultimately succumbed to his illness. Isolated renal involvement of this pathogen is extremely rare in healthy individuals and has poor outcomes. The ubiquitous nature of MM increases the risk of exposure to humans. Comorbidities such as coronavirus disease 2019 and immunosuppressive states are risk factors for the deleterious outcomes of MM. It is unusual for an immunocompetent patient with no underlying conditions to die despite early diagnosis and prompt treatment. This example calls attention to the unpredictable clinical presentation of isolated renal MM. Our case highlights MM as a differential diagnosis in patients with unilateral flank pain and identifies the importance of a prompt clinical diagnosis and treatment due to the rapid progression and poor health outcomes associated with MM infection.

## Introduction

Mucormycosis (MM) is an opportunistic fungal infection of the human body that typically affects immunocompromised individuals but is not limited to immunocompromised hosts [1]. The most common forms of MM are rhinocerebral, gastrointestinal, cutaneous, renal, pulmonary, and disseminated [1]. Disseminated forms of MM resulting in renal involvement have been seen in up to 20% of cases [2]. However, isolated renal involvement has only been reported as case reports [2]. Here, we are presenting a rare case of an isolated renal MM infection in an immunocompetent individual with no known underlying health conditions.

## Case presentation

**Figure 1:**
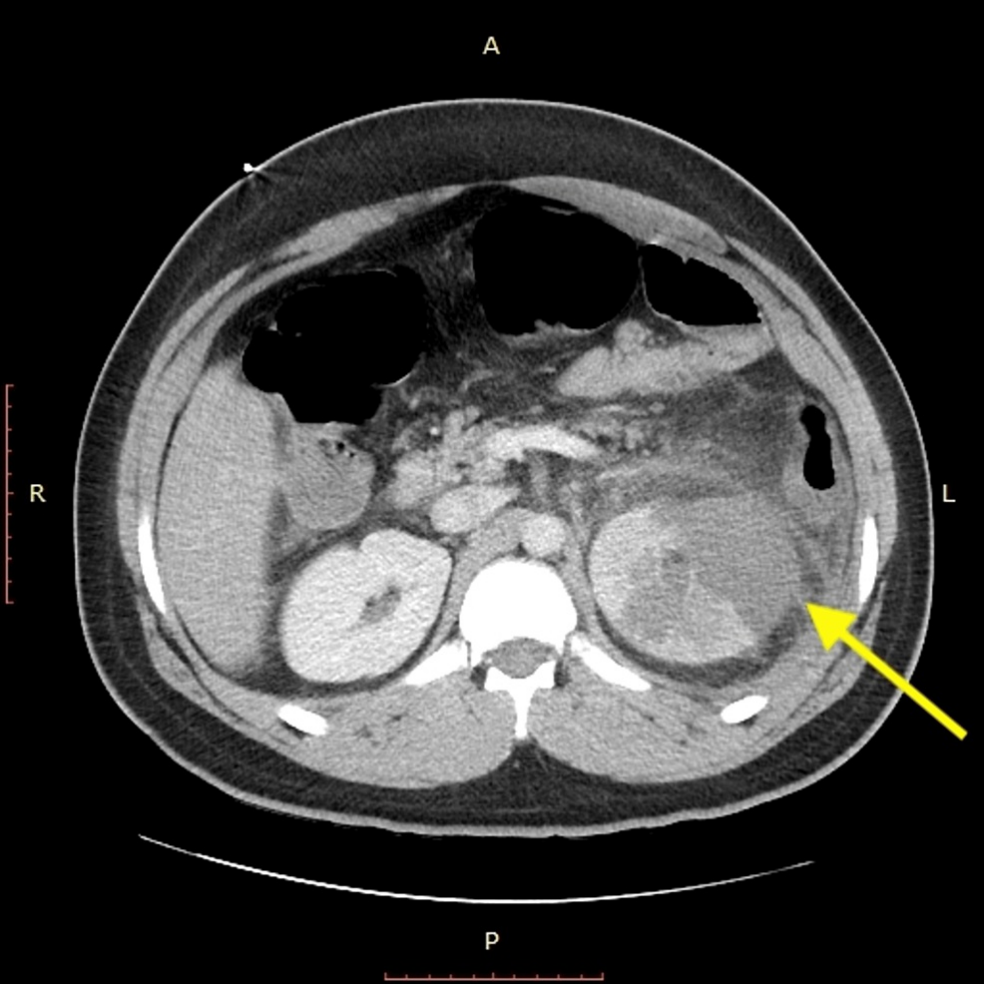
Abdominal computed tomography scan with contrast showing left renal infarction.

A 27-year-old male presented to the emergency room (ER) complaining of left flank pain radiating to his left testicle. A computed tomography (CT) urogram and scrotal ultrasound were unremarkable. It was determined that the likely source of his pain was sciatica, for which he was prescribed Toradol and a steroid pack and discharged home. Over the next two days, his pain intensified and he developed nausea and vomiting. The patient returned to the ER the following day. Vital signs upon admission showed a blood pressure of 165/81 mmHg, pulse of 82 beats/minute, temperature of 36.7°C, respiratory rate of 20 breaths/minute, and oxygen saturation of 98%. A physical examination revealed a tender abdomen in the left lower quadrant and mild tenderness of the left testicle and left inguinal canal. There was no evidence of hernia with the Valsalva maneuver. The patient underwent a CT scan of the abdomen and pelvis with contrast which showed an abnormal hypoperfused left kidney with thickened edematous cortex. Large phlegmon of the left kidney was consistent with the focal expression of pyelonephritis. No kidney stones were detected. Additionally, the scan revealed a 5.3 cm area of decreased attenuation believed to be a possible abscess or infarct on the left kidney (Figure 1).

Differential diagnosis consisted of pyelonephritis and possible renal abscess or infarct. Scrotal ultrasound and CT urogram were unremarkable. Urinalysis was negative for nitrites and leukocyte esterase. Based on the findings of the CT scan, the patient was admitted to the hospital with a diagnosis of left pyelonephritis. The initial blood test results revealed leukocytosis, thrombocytopenia, electrolyte abnormalities, and elevated fasting glucose (Table 1).

**Table 1:** Initial lab values.

Marker	Patient’s value	Reference value
White blood cell count	19 × 10^3^ cells/mm^3^	4.0–10 × 10^3^ cells/mm^3^
Hemoglobin	14.4 g/dL	13.0–16.5 g/dL
Platelet count	143 × 10^3^ cells/mm^3^	150–450 × 10^3^ cells/mm^3^
Sodium	129 mg/dL	135–145 mg/L
Potassium	3.3 mg/L	3.6–5.2 mg/L
Blood urea nitrogen	11.0 mg/dL	6–21 mg/dL
Creatinine	0.8 mg/dL	0.5–1.2 mg/dL
Glucose (fasting)	118 mg/dL	70–99 mg/dL

Intravenous (IV) hydration with normal saline was initiated along with 3.375 g of IV Zosyn every six hours.

**Figure 2:**
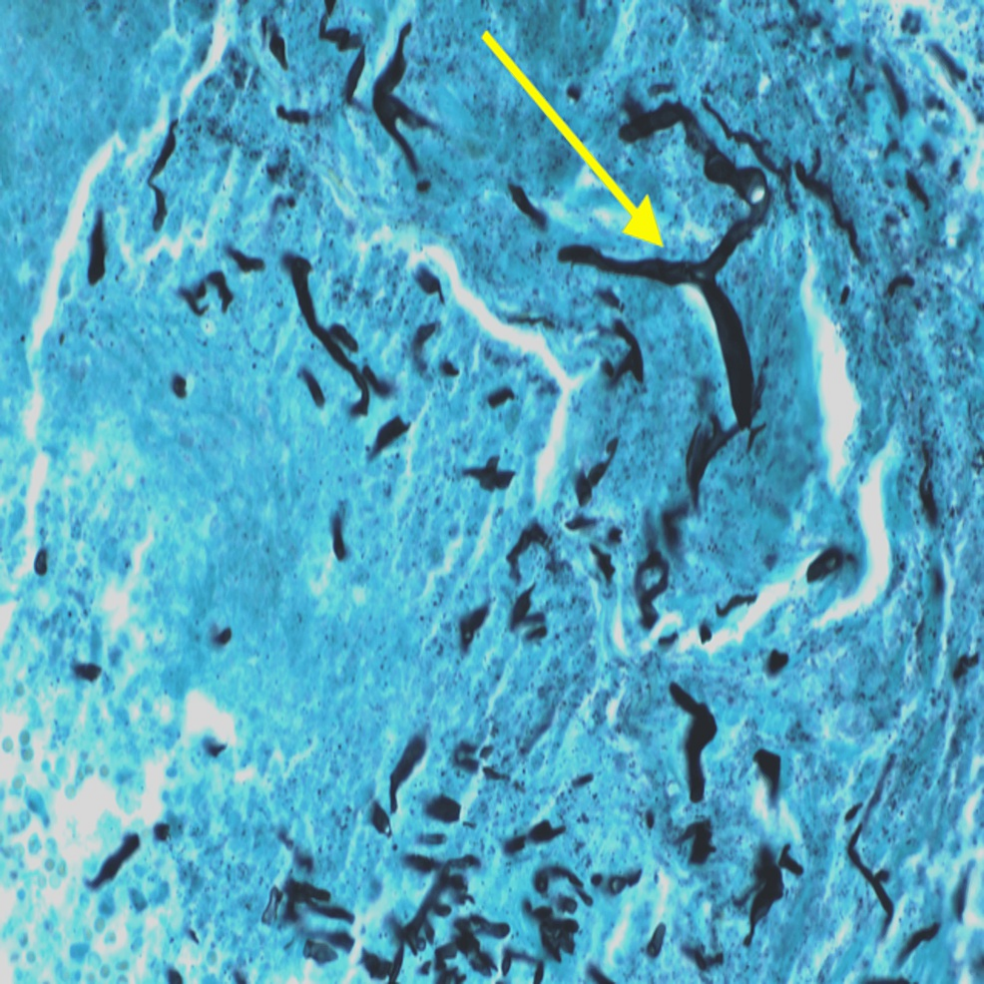
Grocott’s methenamine silver stain of fungal organisms within the renal parenchyma (40×).

The patient’s condition progressed to sepsis. A repeat CT scan identified worsening perinephric edema and decreased perfusion to the majority of the lower half of the left kidney consistent with progressive infarction. At this point, it was determined that the left kidney was the source of the patient’s persistent infection and a nephrectomy was warranted. The patient was taken to the operating room where he underwent a left nephrectomy. Findings during the surgery included an infarcted left kidney and indurated perinephric fat. There was a large inflamed mass that was carefully excised within the perinephric space. A 15-French Blake drain was placed without complications. Pathology of the kidney and left colon revealed MM. Grocott’s methenamine silver (GMS) stain highlighted numerous fungal hyphae with angioinvasion consistent with zygomycosis possibly representing Mucor or Rhizopus. GMS stain revealed non-septate hyphae within the renal parenchyma (Figure 2).

**Figure 3:**
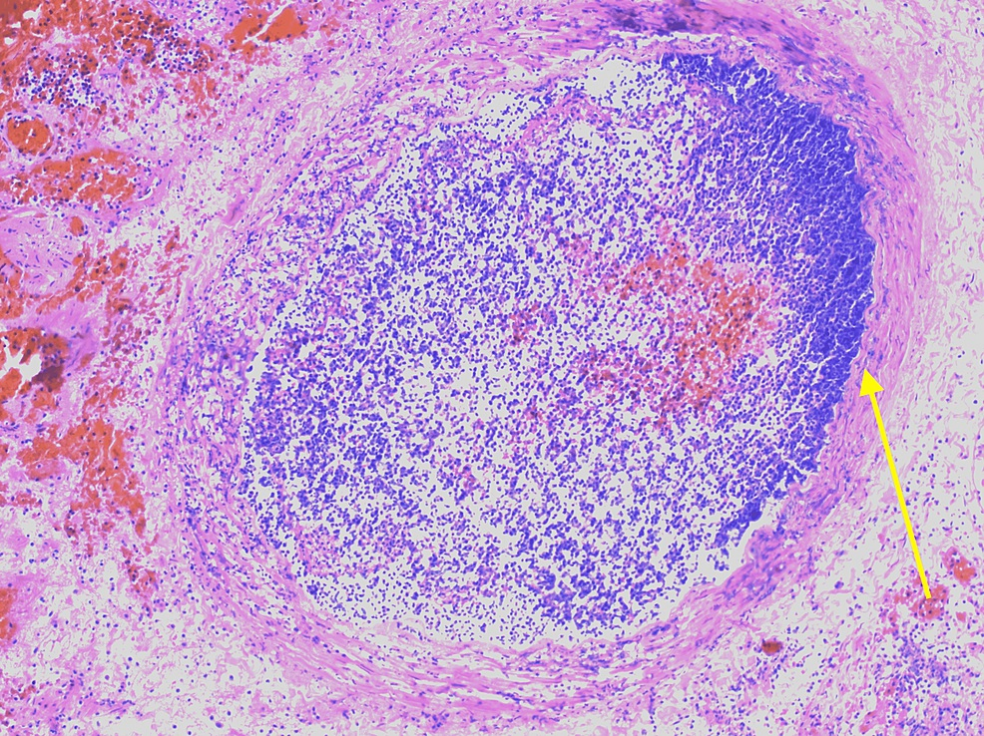
Hematoxylin and eosin picture of the vessel with a necrotizing fungal thrombus (10×).

Hematoxylin and eosin (H&E) stain identified a vessel with a necrotizing fungal thrombus (Figure 3).

At this point in time, 3 mg/kg of IV amphotericin B every day, 500 mg of IV meropenem Flagyl every six hours, and 200 mg of IV isavuconazole every eight hours were initiated.

Five days post-nephrectomy, the patient acquired acute kidney injury and sepsis. CT scan without contrast revealed abnormal mucosal thickening within the splenic flexure and descending colon possibly indicating colitis. The patient underwent diagnostic laparoscopy and exploratory laparotomy. Diagnostic laparoscopy revealed inflammation of the descending colon and splenic flexure. The distal transverse colon, splenic flexure, and proximal descending colon were resected and an end colostomy was performed. An upper 15-French Blake drain was placed over the spleen in the sub-hepatic space, and a lower 15-French Blake drain was placed near the abdominal midline. GMS stain confirmed numerous fungal hyphae indicating the spread of zygomycosis from the left kidney, the site of origin. Four days later, the patient presented with signs of pneumonia, and a repeat CT without contrast of the chest was performed. The CT scan revealed progressive airspace disease with patchy alveolar infiltrates in the upper lobes and dense consolidation in the lower lobes bilaterally. At this point in time, the patient was transferred to a tertiary care center and subsequently passed away.

## Discussion

MM is an angio-invasive fungal infection that carries high morbidity and mortality. Etiology includes filamentous fungi of the class Zygomycetes, order Mucorales containing 11 genera and about 27 species [1,3]. *Rhizopus arrhizus* is the most common infectious agent. *Apophysomyces variabilis* is a major cause in Asia while *Lichteimia *species are predominant in Europe [1]. These fungi are ubiquitous in nature, and human infection is typically acquired via inhalation of spores, ingestion, or traumatic inoculation [4]. Because of the ubiquitous nature of Mucorales and its ability to produce infective spores, there is ample opportunity for exposure to humans. Although mostly seen in Asia, recently, the incidence of MM has increased globally due to new pathogens and susceptible populations [1]. Diabetes mellitus remains the greatest risk factor surrounding MM; however, underlying malignancy, solid-organ and hematopoietic stem cell transplantations, post-tuberculosis, and chronic renal failure have become important risk factors to consider [1,4,5]. Coronavirus disease 2019 (COVID-19) may be a newly emerging risk factor for MM. There has been a recent surge in COVID-19-associated MM. COVID-19 screening was not performed for this patient as this case occurred just prior to the start of the pandemic. Two case review studies analyzed patients diagnosed with COVID-19-associated MM noting that most patients had uncontrolled diabetes and systemic corticosteroid use [6,7]. Furthermore, these cases were associated with poor morbidity and mortality rates [6,7]. While corticosteroid use in COVID-19 has shown to be beneficial in reducing short-term mortality rates and decreasing the need for mechanical ventilation, it is an important reminder that corticosteroid use in COVID-19 patients carries secondary infection risks [8]. Additionally, it is important we recognize that these studies analyzed various disease presentations of MM (rhinocerebral, disseminated, etc.); however, to our best knowledge, no specific cases of isolated renal MM were present. Further research into COVID-19 as a risk factor for MM is required, although initial evidence suggests that a background of diabetes, corticosteroids, and COVID-19 can increase the rates of MM and carries morbid prognoses [6,7]. To what degree COVID-19, corticosteroids, and hyperglycemia are responsible for the increased risk of COVID-19-associated MM has yet to be fully elucidated [6,7].

Renal MM is a rare infectious manifestation presenting with pyelonephritis-like symptoms, such as fever, flank pain, pyuria, and hematuria. Kidney infection is postulated to occur from an ascending urinary tract infection or distant seeding from a subclinical pulmonary infection [9]. Typically, patients with isolated renal MM have risk factors for fungemia such as IV drug use, acquired immunodeficiency syndrome, or IV catheters [10]. Despite this, many cases in Asia have been reported to occur with no known underlying illness [1]. Diagnostic workup involves urinalysis, urine culture, renal ultrasonography, and CT. Imaging can reveal bilaterally enlarged kidneys with thickened renal pelvises and parenchyma infarction due to fungal angioinvasion and subsequent vascular thrombosis [9]. With evidence of renal MM, a renal biopsy can yield broad, irregular, right-angled branching fungal hyphae lacking septations with appropriate staining [9]. Early diagnosis is key to successful treatment of this typically morbid diagnosis. One study noted that early therapy initiation allowed for a 50% survival rate for their 10 isolated renal MM patients [9]. For patients diagnosed with isolated renal MM, survival is estimated to be around 65% [10]; however, mortality increases to almost 100% for patients with bilateral MM infections [9,11]. Management involves surgical debridement, nephrectomy, and antifungal therapy [4,9]. Antifungal pharmacotherapy recommended by the European Confederation of Medical Mycology guidelines of MM advises liposomal amphotericin and notes known MM resistance to echinocandins and certain azoles [4]. Posaconazole and isavuconazole are effective azoles against MM [9]. Non-responding pyelonephritis is typically due to obstructive etiologies such as clots, malignancy, or renal stones; however, fungal etiologies should be considered as appropriate clinical attention and punctual treatment is required for both immunocompetent and immunocompromised individuals with renal MM to improve patient health outcomes.

The presumptions in our case presentation are not without limitations. Although this patient had no known immunodeficiencies, an extensive immunologic analysis was not performed and perhaps would have revealed an underlying immunodeficiency rendering this patient more susceptible to extensive fungal infections. This patient also received Toradol and a steroid pack upon his initial presentation to the ER. Corticosteroids are associated with hyperglycemia and immunosuppressive effects and the combination of these factors can increase infection risk [12]. This represents another limitation as it is unknown how the corticosteroids may have affected the patient’s immunocompetency. Given the short course of medication use, although it is unlikely that the steroid pack alone generated a severe enough immunocompromised state to cause the fulminant infection seen in this patient, it is worth mentioning.

## Conclusions

Our unique and rare clinical presentation describes isolated renal MM in an immunocompetent individual with no known underlying health conditions. Despite prompt diagnosis and therapy with surgical debridement and IV antifungals, the patient’s condition continued to deteriorate and he eventually died. Aggressive multimodality therapy continues to be the first-line treatment plan for survival. Therefore, MM should be part of the differential diagnosis. Although rare, it is serious with a poor prognosis.
